# Fast and bright spontaneous emission of Er^3+^ ions in metallic nanocavity

**DOI:** 10.1038/ncomms8080

**Published:** 2015-05-05

**Authors:** Jung-Hwan Song, Jisu Kim, Hoon Jang, In Yong Kim, Indra Karnadi, Jonghwa Shin, Jung H. Shin, Yong-Hee Lee

**Affiliations:** 1 Department of Physics, Korea Advanced Institute of Science and Technology (KAIST), Daejeon 305–701, South Korea; 2 Department of Materials Science and Engineering, Korea Advanced Institute of Science and Technology (KAIST), Daejeon 305–701, South Korea; 3 Graduate School of Nanoscience and Technology, Korea Advanced Institute of Science and Technology (KAIST), Daejeon 305–701, South Korea

## Abstract

By confining light in a small cavity, the spontaneous emission rate of an emitter can be controlled via the Purcell effect. However, while Purcell factors as large as ∼10,000 have been predicted, actual reported values were in the range of about 10–30 only, leaving a huge gap between theory and experiment. Here we report on enhanced 1.54-μm emission from Er^3+^ ions placed in a very small metallic cavity. Using a cavity designed to enhance the overall Purcell effect instead of a particular component, and by systematically investigating its photonic properties, we demonstrate an unambiguous Purcell factor that is as high as 170 at room temperature. We also observe >90 times increase in the far-field radiant flux, indicating that as much as 55% of electromagnetic energy that was initially supplied to Er^3+^ ions in the cavity escape safely into the free space in just one to two optical cycles.

## Abstract (web summary)

The Purcell effect predicts a spontaneous emission rate enhancement of several orders of magnitude, but experimental demonstrations have been much lower. Here, Song *et al.* show emission enhancement of Er^3+^ ions in a metallic nanocavity with a 170 Purcell factor at room temperature and 55% extraction efficiency.

## Graphical abstract

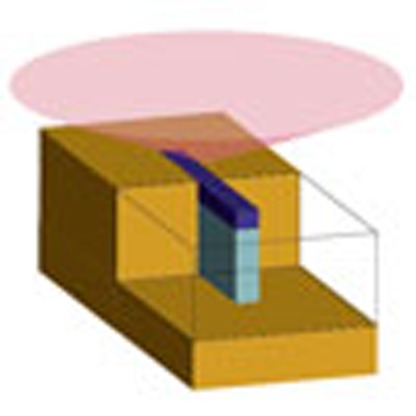

Ever since Purcell's 1946 theory on the spontaneous emission probabilities at radio frequencies^1^, controlling spontaneous emission at optical frequencies has been the subject of intense research in the photonics community, and by now, many different forms of resonant cavities have been investigated with the goal of enhancing the spontaneous emission rate of an emitter in the cavity^2^,^3^,^4^,^5^,^6^,^7^,^8^,^9^,^10^,^11^,^12^,^13^,^14^,^15^,^16^,^17^,^18^,^19^,^20^,^21^,^22^. Achieving a strong enhancement, or a large Purcell factor, would not only facilitate the development of many novel devices that would benefit from fast, bright-light emission^3^,^4^,^5^, but also provide a valuable tool in investigating the fundamentals of photon-matter interaction^6^. In many occasions, the Purcell factor was given to be 
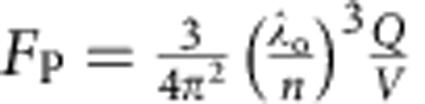
, where *λ*_o_/*n* is resonance wavelength in the cavity, *Q* is the quality factor of the cavity and *V* is the mode volume, and much effort was directed to increasing the *Q*/*V* ratio, oftentimes using ultra-high *Q* cavities^7^. However, the above expression is valid only for an emitter that is perfectly matched to the cavity in every way—spectrally, spatially and polarization wise^8^, with the full expression given by 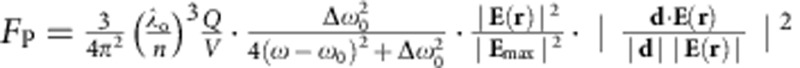, where *ω*_0_ and **E**(**r**) are the angular frequency and electric field of the resonance, respectively, and **d** is dipole moment of an emitter^9^. For high-*Q* cavities, such a perfect match to a realistic emitter is difficult, especially at room temperature. Consequently, despite ‘predictions' of Purcell factors in excess of 10,000, the values actually obtained experimentally were much lower, typically in the range of 3–30 (refs 10, 11, 12, 13, 14). A Purcell factor of 75 had been reported for a quantum well in a photonic crystal cavity, but only at cryogenic temperatures^3^.

Another possible approach is the use of metallic nanocavities^15^,^16^,^17^,^18^,^19^,^20^,^21^,^22^,^23^,^24^,^25^. The *Q*-factors of metallic nanocavities are quite modest, but the mode volume can be made to be very small, thus achieving a high *Q*/*V* ratio. The reported values of Purcell factors, however, still remained quite low, in the range of 10–30 only. Furthermore, the presence of metal can introduce Joule losses, which result in an increase in the non-radiative decay rate and a reduction in the far-field radiation intensity from such a cavity. This not only makes quantitative analysis of decay rate difficult, but also reduces the usefulness of such a cavity in practical applications.

In this paper, we report on the enhancement of 1.54-μm emission of Er^3+^ ions placed in a very small metallic nanocavity. Er^3+^ ions were chosen for their ^4^I_13/2_→^4^I_15/2_ intra-4f transition at the technologically important wavelength of 1.54 μm. As this is a parity-forbidden transition of core electrons, its luminescence lifetimes can be longer than 10 ms. Such a long luminescence lifetime allows for a direct measurement of a Purcell factor well over 1,000 since the temporal response of a typical avalanche photodetector is in the range of nanoseconds. In addition, as this is a core-level transition of dopant atoms immobilized in a host material such as SiO_2_, issues such as surface recombination and carrier diffusion that complicate analysis of data from semiconductor-based emitters such as quantum dots do not arise. A trench-type gold nanocavity was chosen for its small mode volume and ease of fabrication. The cavity structure was systematically designed not only to increase the *Q*/*V* ratio, but also to provide high degree of matching between Er^3+^ ions and the cavity, both spectrally and spatially. In addition, care was taken to enhance the extraction of light from the cavity as well. On the basis of a systematic investigation of many photonic properties, together with comparison with full three-dimensional (3D) numerical simulations, we demonstrate an unambiguous Purcell factor that is as high as 170 at room temperature, in an excellent agreement with the predicted value of 220. We also observe >90 times increase in the far-field radiant flux, indicating an extraction efficiency as high as 55%, due to extremely fast radiation of electromagnetic energy from Er^3+^ ions in the cavity into the free space in just 1–2 optical cycles before experiencing Joule losses can occur.

## Results

### Cavity design and fabrication

SiO_2_ film (200-nm thick) with a 40-nm-thin Er-doped layer was deposited on a quartz substrate using reactive ion beam sputtering method [Method]. The Er^3+^ density was 10^20^ cm^−3^, which corresponds to one Er^3+^ ion per 10 nm^3^. In the silica matrix, the Er^3+^ emission near the 1.54-μm is inhomogeneously broadened and is also subject to non-radiative recombination processes. In bare SiO_2_, the apparent total decay rate (*γ*_0_^tot^=1/*τ*_0_^tot^) of Er^3+^ ions is measured to be (10 ms)^−1^ (see Supplementary Fig. 1 and Supplementary Note 1), which is a sum of radiative (*γ*_0_^r^) and non-radiative (*γ*_0_^nr^) decay rates. We separate these competing recombination processes experimentally by overcoating the Er-doped SiO_2_ film with high-index Si layers of varying thickness, thereby controlling the local density of states at the position of the Er^3+^ ions (see Supplementary Fig. 2 and Supplementary Note 2). The intrinsic radiative lifetime *τ*_0_^r^ (=1/*γ*_0_^r^) is found to be 21 ms, in good agreement with previous reports^26^.

**Figure 1:**
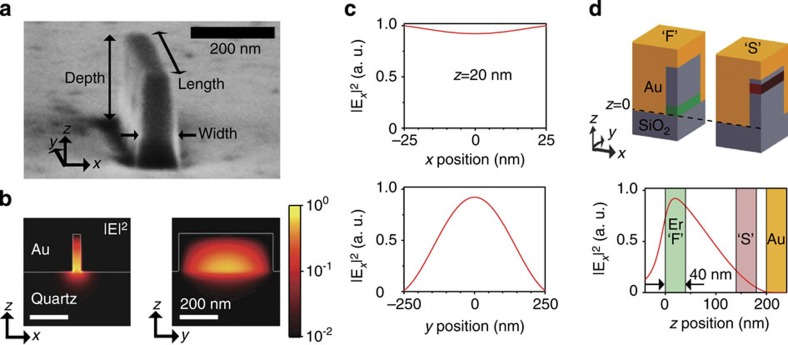
Au nanotrench with Er^3+^. (**a**) Fabricated nanotrench before gold deposition. (**b**) |E|^2^ computed by 3D-FDTD at 1,540 nm. Width, length and depth are set to be 50, 500 and 200 nm, respectively. (**c**) |E_*x*_|^2^ along the antinodal plane (*z*=20 nm). The magnitude was normalized by the maximal value at |*x*|=25 nm. (**d**) Cross-sectional view of an Au nanotrench. Positions of Er-doped SiO_2_ layer in the fast (F) and slow (S) samples are indicated by green and red colours, respectively. |E_*x*_|^2^ along the *z* axis. Thickness range of the Er-doped layers in F and S samples are also indicated in green and red colours, respectively.

The Au nanotrench used in this paper has a simple rectangular geometry of a long narrow rectangular trench in a thick Au layer that is filled with SiO_2_. Despite the small dimensions, accurate control over cavity dimensions and strucure is quite easy due to the inverted nature of fabrication process, where a narrow SiO_2_ mesa containing a thin Er^3+^ doped layer is first fabricated as shown in Fig. 1a, and then covered with a thick Au layer, thus forming a ‘trench' in the Au layer. This ensures that Er^3+^ ions are located inside the Au nanotrench only; those outside the trench are completely etched away during the fabrication process (see Supplementary Fig. 3 and Supplementary Method 1). The resonance wavelength of the cavity can be controlled by varying the trench (that is, the original SiO_2_ mesa) width, length and depth. As Fig. 1b,c shows, the fundamental plasmonic mode whose electric field oscillates along *x* direction shows a simple electric field distribution that is easy to analyse. The photon density along the 50-nm-wide *x* direction stays almost constant. Along the longer *y*- and *z* directions, it closely approximates the cosinusoidal functional shape. Such a smooth mode profile allows one to observe significant amount of Purcell enhancement even when averaged over emitters that are spread across the entire optical mode, with robustness against experimental errors (see Supplementary Fig. 4 and Supplementary Note 3). When the trench dimension is 50 × 500 × 200 nm^3^, the nanocavity has a mode volume of 7 × 10^−4^ (*λ*/*n*)^3^ and a fundamental plasmonic resonance near 1,540 nm with a *Q* factor of ∼5. We also note that at this length scale, no severe degradation of the modal properties due to plasmonic nonlocal effect is expected^27^.

For comparison purposes, two different Er^3+^-doped films (fast ‘F', slow ‘S' samples) were prepared and studied. In the F sample, Er^3+^-doped layer was located at the opening of the trench (0<*z*<40 nm) where the antinode of the fundamental plasmonic mode will be formed. For the S sample, Er^3+^-doped layer was located deeper in the trench (140 nm<*z*<180 nm) where the modal field is weaker (Fig. 1d). In all cases, the local Er^3+^ density was kept the same at 10^20^ cm^−3^, such that in both F and S samples, about 10^5^ ions participate in the spontaneous emission processes. The trench length was varied between 360 and 660 nm, while the trench width and depth were kept constant at 50 and 200 nm, respectively.

**Figure 2:**
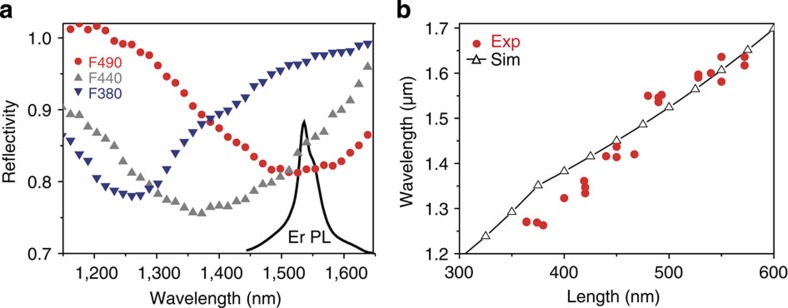
Spectral characteristics of Au nanotrenches. (**a**) Reflectivity spectra of fabricated samples of different lengths for fixed width (50 nm) and depth (200 nm), normalized with respect to a gold coated reference. Only a single Au nanotrench is illuminated using an *x*-polarized broadband tungsten-halogen source. A 490-nm-long nanotrench shows a resonance near 1,540 nm that matches the measured Er emission spectrum (black solid curve). (**b**) Structural tuning of fabricated Au nanotrenches. Triangular dots are from the 3D-FDTD simulations.

The normal reflection spectra of Au nanotrenches of different lengths are shown in Fig. 2a. Broad resonance dips indicative of plasmon resonance can be seen. Figure 2b compares the measured resonance wavelengths with calculated plasmon resonance wavelengths at different trench lengths. Excellent agreement is found, confirming that the resonances are indeed due to excitation of the plasmon mode in the cavity. The quality factors of the resonances, obtained from the width of the resonance dips, were ∼5 for all resonances.

### Fast spontaneous emission

**Figure 3:**
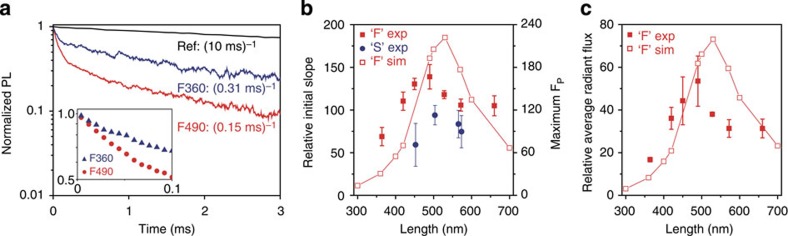
Fast and bright spontaneous emission. (**a**) Time-resolved photoluminescence (PL) traces of 490-nm-long (red), 360-nm-long (blue) and reference (black) samples, respectively. Transient behaviours during the initial 0.1 ms are shown separately in the inset. (**b**) Relative initial slope of Er^3+^ inside the Au nanotrenches for the F (red square) and S (blue circle) samples. Hollow square symbols are theoretical predictions of the relative initial slope for the F samples. (**c**) Relative average radiant flux of Er^3+^ in F samples compared with that in the reference sample. The difference of polarization-dependent collection efficiency between F and reference samples is taken into consideration. Hollow symbols are corresponding theoretical predictions.

Figure 3a shows the decay traces of Er^3+^ luminescence from the reference film, an F sample with a cavity length of 490 nm and an F sample with a cavity length of 360 nm. We find that the decay rate from cavities is much higher, as expected. Furthermore, the decay rate from the 490 nm long cavity, whose plasmon resonance coincides with the Er^3+^ luminescence wavelength, is much higher than that from the 360 nm long cavity, whose plasmon resonance occurs near 1,250 nm and is thus detuned from the Er^3+^ luminescence, consistent with the effect of Purcell enhancement on the light emission from Er^3+^ ions.

For a more quantitative analysis, we concentrate on the initial slope of the decay trace near *t*=0, as it represents –[∑*γ*^2^(**r**_i_)]/[∑*γ*(**r**_i_)] in the ensemble of many randomly-polarized Er^3+^ ions decaying at different rates, where *γ*(**r**_i_) is position-dependent total decay rate of an Er^3+^ ion placed at a position **r**_i_ and the summation is over all the Er^3+^ ions in an Au nanotrench (see Supplementary Fig. 5 and Supplementary Note 4). The initial slope also has the advantage of providing the highest signal/noise ratio. The slow tail of the decay trace is experimentally confirmed to stem from very weak low-frequency system noises. The relative initial slopes measured from F samples with cavities of different lengths, normalized to the intrinsic radiative decay rate (*γ*_0_^r^) from the reference film, are summarized in Fig. 3b. Also shown for comparison are the predicted values for the relative initial slopes that were calculated based on the Er^3+^ concentration profile and the optical mode profile (see Supplementary Note 5). Overall, the agreement is very good. The maximum relative initial slope is 140, corresponding to a Purcell factor of 170 at the mode maximum, is obtained from the 490 nm long cavity, in good agreement with the predicted maximum initial slope of 180, corresponding to a Purcell factor 220 at the mode maximum. For comparison, we also show the relative initial slopes measured from the S samples. They are always lower, consistent with the location of their Er^3+^ ions deeper in the trench, where the modal field is weaker. In addition, we confirmed that the measured photoluminescent signal integrated over all the randomly oriented ions in the metallic nanocavity is nearly *x* polarized with a polarization extinction ratio of >100, in good agreement with theory (see Supplementary Fig. 4e).

### Bright spontaneous emission

However, such enhanced decay rate by itself is not a sufficient proof of Purcell effect. In an ideal situation, the luminescence intensity under the saturation pumping condition should be Purcell enhanced by the same factor as the decay rate as well (see Supplementary Fig. 6 and Supplementary Note 6). Indeed, controlling spontaneous emission makes practical sense only when the accelerated radiative recombination is the dominant energy flow channel. However, when we employ metallic nanocavities, we also inevitably introduce Joule loss stemming from electron collisions. Thus, we need to investigate whether we can extract the electromagnetic energy stored in the metallic nanocavity with an efficiency close to the intrinsic quantum efficiency (=0.5) of Er^3+^ ions embedded in bare SiO_2_. Figure 3c shows the experimentally measured average relative radiant flux enhancement from F samples with cavities of different lengths, normalized to that from the reference film. Also shown are the predicted values, based on calculations that include all possible effects including the Purcell enhancement, different extraction efficiencies, collections efficiencies and Joule losses (see Supplementary Table 1 and Supplementary Note 7). Again, the agreement is very good. A maximum enhancement factor of average radiant flux is 54, corresponding to an enhanced radiant flux of 93 at the mode maximum, is obtained from the 490-nm-long cavity, in good agreement with the predicted maximum enhancement of 72, corresponding to enhanced radiant flux of 124 at the mode maximum.

It should be noted here that the calculated values are pure predictions without any fitting parameter. The fact that a single set of calculations can correctly predict the observed enhancements of both decay rate and radiant flux is a strong indication that we have indeed achieved hitherto highest Purcell factor of 170.

### Energy loss channels

The difference between the enhancement factors of decay rate and radiation flux occurs because the electromagnetic energy stored in the metallic nanocavity can be dissipated through several different loss channels. The radiative channel constitutes the useful output and the non-radiative Joule loss channels are composed of the contribution related to surface and bulk plasmons. We define the normalized coupling efficiency of the radiative channel (*ρ*_rad_) to be the ratio of the integrated electromagnetic flux propagating into the upper hemisphere to the total power supplied to the dipole emitters coupled to the cavity mode of interest. On the basis of this definition, measured far-field relative average radiant flux of 54 corresponds to a coupling efficiency of 0.55 (=93/170), meaning that 55% of the photons emitted from the Er^3+^ are released before being dissipated in the metal.

**Figure 4:**
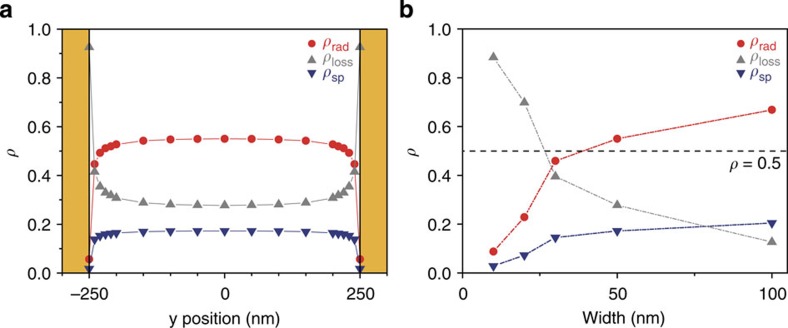
Energy-coupling channels. (**a**) Normalized coupling efficiency of propagating radiation (*ρ*_rad_), Joule loss (*ρ*_loss_) and surface plasmon (*ρ*_SP_) calculated by 3D-FDTD computations with *x*-polarized light. The width, length and depth of the metallic nanotrench are 50, 500 and 200 nm, respectively. (**b**) Normalized coupling efficiency as a function of the width. The dipole source is placed at the centre, *x*=*y*=*z*=0. The length is slightly modified to keep the resonant wavelength at 1,540 nm. The black dashed line along *ρ*=0.5 is drawn as an eye guide for the intrinsic quantum efficiency of Er^3+^.

Figure 4a,b shows the detailed theoretical analyses of such loss mechanisms performed by 3D finite-difference time-domain (3D-FDTD) methods (see Methods). For almost all the ions except those in close proximity to the metallic surfaces, the normalized coupling efficiencies stay nearly unchanged. The large *ρ*_loss_ near the metallic boundaries is partly attributed to the interactions between the source dipole and the image dipole in the metal^28^,^29^. However, for Au nanotrenches with width >50 nm, electromagnetic energy emitted from the ions in the middle of the cavity find their way out in the form of radiation with efficiency that is >50% (Fig. 4b). In case of the nanotrench with a width of 50 nm, the calculated extraction efficiency (=*ρ*_rad_) is about 55%, in good agreement with the experimentally measured efficiency of 55%, further strengthening our estimation of 170 for the value of the maximum Purcell factor achieved in the nanocavity.

Still higher Purcell factor may be obtained by using a narrower trench to reduce the mode volume. However, as Fig. 4b shows, doing so significantly reduces the extraction efficiency. Thus, the trench width of 50 nm represents an optimum trade-off between enhancing the Purcell factor and the extraction efficiency. We note, however, that a better trade-off may be possible by using a metal with lower resistivity^24^.

In summary, we report on achieving fast and bright spontaneous emission from Er^3+^ ions embedded in the metallic nanocavity. We find that the Er^3+^ ions placed in the metallic nanocavity generate an ensemble-averaged PL with an intensity that is >50 times brighter, with an initial slope that is >140 times faster, than those in bare SiO_2_, under saturation pumping conditions. These values, together with their comparison with theoretical values, enable us to demonstrate unambiguously a hitherto largest achieved overall Purcell factor of 140, corresponding to a local Purcell factor of 170 at the mode maximum. A high extraction efficiency of 55% is achieved at the same time due to the low-*Q* nature of the metallic resonators. We believe that the metallic nanocavity can make a competitive platform that enables the fast and bright spontaneous emission for applications such as fast and efficient light-emitting diodes and efficient single photon sources.

## Methods

### Optical characterization

Samples were pumped with a 980-nm InGaAs laser using an objective lens (N.A.=0.5). PL signals within the bandwidth of 1,530–1,550 nm were filtered and fed into an infrared-photomultiplier tube after the pump beam was carefully blocked. The pump laser was turned on and off at a rate of 7 Hz and the decaying PL traces were integrated over 10,000 times to improve the signal to noise ratio of the weak optical signal. To avoid unnecessary complications, a single nanotrench was selectively pumped and monitored throughout the PL experiment. For all the PL experiments, the state of polarization of the pump beam is set perpendicular to the length of the nanotrench (*x* direction). The *y*-polarized pump beams do not penetrate well into the depth of metallic nanotrench and the resultant PL intensity is negligibly weak. For reflectivity spectra measurement, InGaAs laser was replaced by collimated tungsten-halogen lamp beam. The beam was modulated by an optical chopper at ∼200 Hz and the reflected signals were amplified by lock-in amplifier.

### Er^3+^ deposition conditions

We deposit a 200-nm-thick SiO_2_ slab that contains 40-nm-thick Er-doped layer on quartz substrate by multi-target, reactive ion beam sputtering deposition method. We adopt two-target alternation system; one target is a Si wafer and the other is a Si wafer with an Er piece attached on the top. By changing targets, we are able to select where the Er-doped layer is to be embedded, with nm-scale precision precisely. Er-free SiO_2_ layer is deposited by using the Si target, while the Er-doped SiO_2_ layer is deposited by using the Si target with the Er piece. Base pressure is 2 × 10^−7^ Torr and Ar and O_2_ gases are injected at the rates of 1 and 4 s.c.c.m., respectively. 600-eV-Ar^+^ ions accelerated from an ion gun sputter the targets. After deposition, all the samples are treated by rapid thermal annealing at 950 °C for 20 min in Ar gas environment. We analyse the composition of the Er-doped SiO_2_ layer by Rutherford backscattering spectroscopy, which identifies 33.3 at. % of Si, 66.5 at. % of O and 0.2 at. % of Er. The volume density of Er^3+^ ions in the Er-doped layer is 1.0 × 10^20^ cm^−3^.

### 3D-FDTD simulation conditions

The whole 3D-FDTD computational domain was defined as a cuboid volume of 1,280 × 2,560 × 1,280 nm^3^ divided by uniformly partitioned 4-nm spatial grids and 6.7 as temporal grids. To avoid unphysical reflection at the simulation boundaries, we defined 160-nm-thick convolutional perfectly matched layers at each facet. Gold was described by the lossy Drude model that provides valid dielectric constants of gold from 600 to 2,000 nm spectral range. There are Drude parameters in the following formula, *ɛ*(*ω*)=*ɛ*_∞_−*ω*_p_^2^/(*ω*^2^+*iγω*). We set the background dielectric constant (*ɛ*_∞_), plasma frequency (*ω*_p_) and collision frequency (*γ*) to be 10.48, 1.376 × 10^16^ and 1.177 × 10^14^ s^−1^, respectively.

### Energy flow analysis

We calculated the normalized coupling efficiency of a dipole source in an Au nanotrench to propagating radiation, Joule loss, and surface plasmon. At first, we calculated the total power dissipation of the dipole source (*P*_tot_) by taking the inner product between the local electric field and current density at local position of the dipole source. Integration of Poynting vector over a rectangular enclosure (see Supplementary Fig. 7a) gives the propagating power (*P*_prop_), which amounts to the sum of the propagating radiation flux (*P*_rad_) and surface plasmon flux (*P*_SP_). By the principle of energy conservation, the subtraction of *P*_prop_ from *P*_tot_ results in the power dissipation by Joule loss (*P*_loss_). Knowing that the *P*_rad_ is also obtained by the integration of far-field distribution over total solid angle, we also extracted the surface plasmon flux (*P*_SP_) by the subtraction of *P*_rad_ from *P*_prop_. Since the length of the rectangular enclosure was 800 nm that is quite shorter than the propagation length of surface plasmon at 1,540 nm (∼10 μm), we neglected the amount of Joule loss of propagating surface plasmon inside the enclosure. The normalized coupling efficiencies to each energy flow channel in Fig. 4a,b were calculated by *ρ*_rad_=*P*_rad_/*P*_tot_, *ρ*_loss_=*P*_loss_/*P*_tot_, *ρ*_SP_=*P*_SP_/*P*_tot_, respectively.

## Author contributions

Y.-H.L. conceived the basic idea. J.-H. Song and J. Shin actualized the basic idea by designing the structures. J.K. and I.Y.K. deposited Er/SiO_2_ layer by sputtering. J.-H. Song made chromium mask through e-beam lithography and liftoff processes. J. Kim performed RIE processes. J.-H. Song performed theoretical analyses including full-field 3D-FDTD simulations and characterized fabricated samples. H. Jang made signal processing programs and characterized focused excitation beam by knife-edge measurement. J.-H. Song, J.H. Shin and Y.-H.L. wrote the manuscript. All authors have been agreed to be responsible with the content of the publication.

## Additional information

**How to cite this article**: Song, J-H. *et al.* Fast and bright spontaneous emission of Er^3+^ ions in metallic nanocavity. *Nat. Commun.* 6:7080 doi: 10.1038/ncomms8080 (2015).

## Supplementary Material

Supplementary Information Supplementary Figures 1-7, Supplementary Table 1, Supplementary Notes 1-7, Supplementary Methods and Supplementary References
