# Supplementary material for: Fast and bright spontaneous emission of Er3+ ions in metallic nanocavity
Source: Nat Commun. 2015 May 5;6:7080. doi: 10.1038/ncomms8080 (PMC4432579; doi:10.1038/ncomms8080)
Supplement: Supplementary Information — Supplementary Figures 1-7, Supplementary Table 1, Supplementary Notes 1-7, Supplementary Methods and Supplementary References [file ncomms8080-s1.pdf]

Supplementary Figures

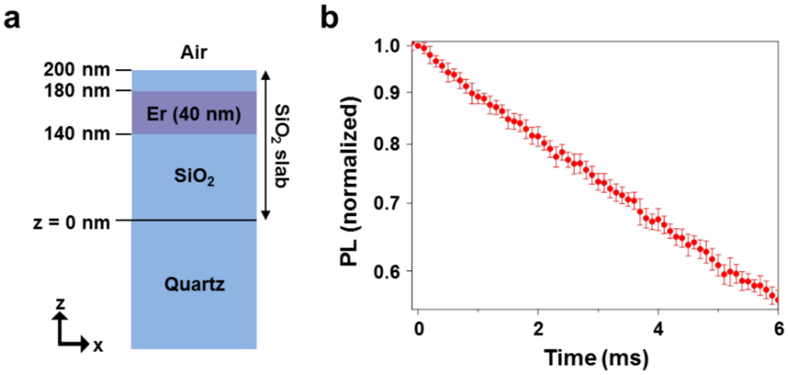

Supplementary Figure 1 | Intrinsic total decay rate of Er^{3+} ions a, Schematic of reference sample. **b,** Averaged time-resolved PL trace of randomly selected 10 spots of the reference sample. The symbol and error bar indicate the averaged PL intensity and standard deviation for the 10 spots, respectively.

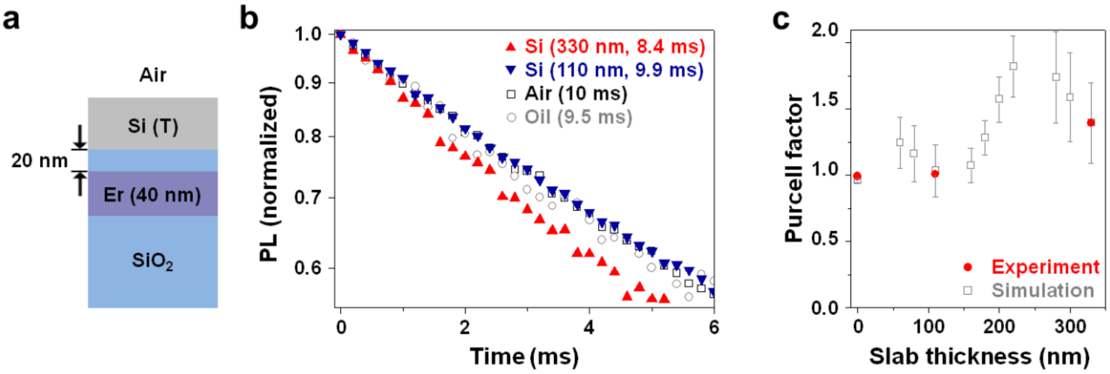

Supplementary Figure 2 | Intrinsic radiative decay rate of Er^{3+} ions a, Schematic of sample. **b,** Time-resolved PL traces for different ambient media conditions. **c,** Purcell factor determined by experiments (red symbols) and that calculated by 3-D FDTD simulations (gray symbols). Calculated Purcell factor in the simulation is averaged over 40-nm-thick range of Er-doped layer.

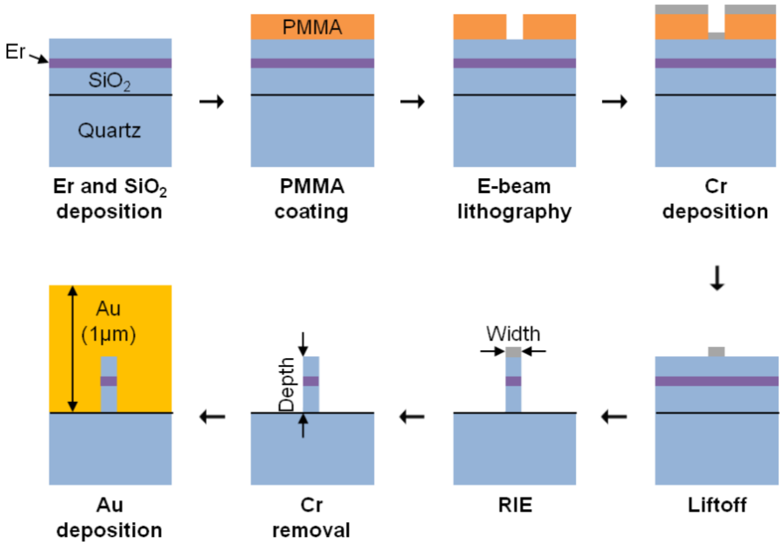

Supplementary Figure 3 | Fabrication process for an Au nano-trench The Au layer thickness is not to scale.

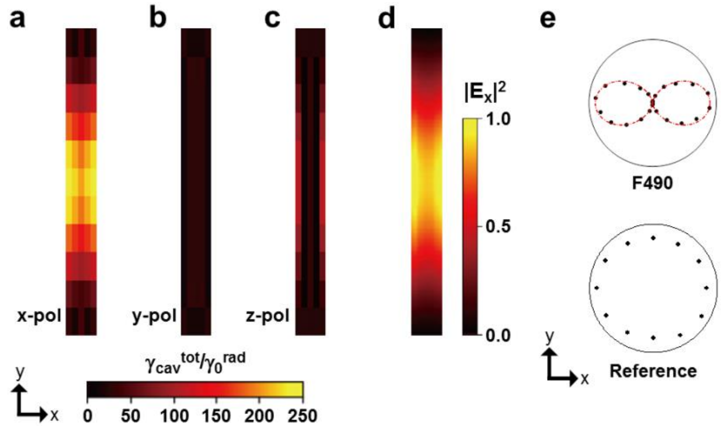

Supplementary Figure 4 | Polarzation-dependent spatial distribution of total decay rate enhancement a-c, Calculated total decay rate enhancement of the *x*-polarized dipole source (a), *y*-polarized dipole source (b), and *z*-polarized dipole source (c) in 500-nm-length Au nano-trench. **d,** Spatial mode profile of $|E_x|^2$ at the same plane calculated by 3D-FDTD simulations. **e,** PL intensity vs. polarization angle for the F490 Au nano-trench and reference sample.

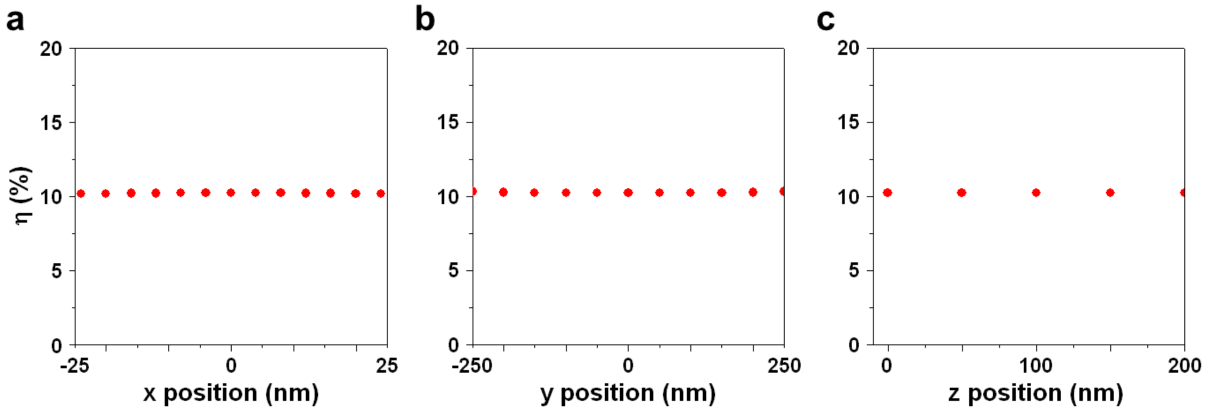

Supplementary Figure 5 | Collection efficiency of emitters in Au nano-trench a, Calculated collection efficiency of an *x*-polarized dipole source inside an Au nano-trench (500-nm length) as scanning the *x* position. **b,** Collection efficiency as varying the *y* position. **c,** Collection efficiency as varying the *z* position.

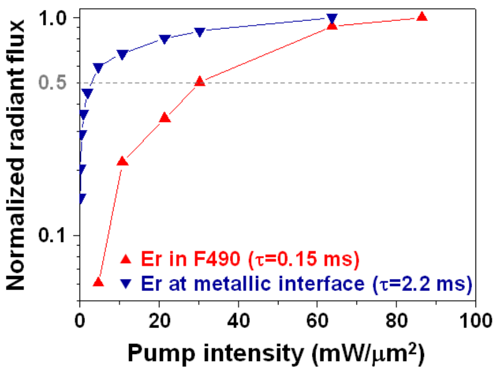

Supplementary Figure 6 | Normalized radiant flux of PL vs. pump intensity

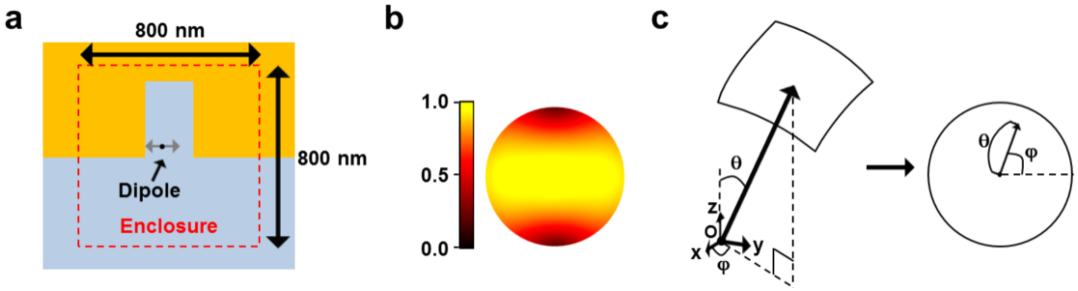

Supplementary Figure 7 | Near-to-far-field transformation method a, Geometry of a rectangular enclosure (red dashed rectangle) for phasor calculations. **b,** Far-field distribution of a dipole source (*x*-polarized) coupled to the Au nano-trench. **c,** Coordinate transformation from the spherical to 2-D Cartesian coordinate.

Supplementary Tables

Supplementary Table 1 | Light extraction efficiency of Au nano-trench

**Cavity length (nm)**	300	360	400	420	490	530	600	700
**Extraction efficiency (%)**	39	46	50	51	55	56	58	59

Supplementary Notes

Supplementary Notes 1 | Decay rate of Er^{3+} in bare SiO_2 matrix

We set 40-nm-thick Er-doped SiO_2 layer embedded in 200-nm-thick SiO_2 slab on quartz substrate as a reference sample as depicted in Supplementary Figure 1a. The Er-doped layer is sputter-deposited in the depth range from z=140 to z=180 nm. This is the same film that was used to fabricate the fast “F” samples. This is a good choice as a reference sample since the Purcell factor in the Er-doped position is ~1 (=0.96) for the x- and y-polarized dipole sources. Purcell factor of the z-polarized dipole (=0.43) rarely contributes to measurement data; the collection efficiency for the z-polarized dipole source is ~30 times smaller than that for the x- and y-polarized dipole sources.

To check the intrinsic total decay rate as well as the local homogeneity of the Er^{3+} ions, we randomly selected 10 spots and plot their statistical average decay trace in Supplementary Figure 1b. Single exponential decay feature is observed with the average decay time constant of 9.8 ms. Only 2% of fluctuation supports the homogeneity of the Er^{3+} ensembles over regions including Au nano-trench samples and the reference sample. This, near $(10\ \text{ms})^{-1}$ total decay rate constitutes of radiative (γ_0^{r}) and nonradiative (γ_0^{nr}) decay rates, which are decomposed by the method of modifying LDOS in the next section.

Supplementary Notes 2 | Intrinsic radiative decay rate of Er^{3+}

In a specific situation where LDOS is controlled, it is possible to extract radiative decay rate from total decay rate. This technique was firstly adopted by Polman et al. when they reported the radiative decay rate of Er^{3+} embedded in bare SiO_2 matrix is $(20\ \text{ms})^{-1}$ [1]. We also controlled the LDOS at the position of Er-doped layer in the reference sample by changing the ambient media or depositing silicon slab as indicated in Supplementary Figure 2a.

The decay traces with different ambient conditions plotted in Supplementary Figure 2b manifest the influence of modified LDOS. Only minute change in the decay rate is observed when the reference sample is covered by index matching oil (n=1.5). Discernable increase of the decay rate to $(8.4\ \text{ms})^{-1}$ is observed when the 330-nm-thick high-index (n=3.4) silicon slab is deposited. For thinner silicon slab (110 nm), the decay rate is again decreased to $(9.9\ \text{ms})^{-1}$, which is consistent to the theoretical predictions by 3D-FDTD simulations; theoretically calculated Purcell factor (F_P) for the 330-nm-thick silicon slab is 1.40 whereas that for the 110-nm-thick slab is 1.03 only.

In experiments, F_P can be determined by a formula of $F_P(T) = (\gamma^{tot}(T)-\gamma_0^{nr})/\gamma_0^{r}$, which are plotted as a function of silicon slab thickness (T) in Supplementary Figure 2c (red symbols). γ^{tot} is the total decay rate of the reference sample with the silicon slab while γ_0^{r} and γ_0^{nr} are the radiative and nonradiative decay rate without the slab, respectively. The radiative decay rate (γ_0^{r}) and the nonradiative decay rate (γ_0^{nr}) are determined by fitting the experimentally determined F_P to that of theory. Allocation of $\gamma_0^{r} = (21\pm4\ \text{ms})^{-1}$ and $\gamma_0^{nr} = (19\pm3\ \text{ms})^{-1}$ gives the best and is also consistent to the previous study [1].

Supplementary Notes 3 | Position and polarization dependence of LDOS of an Er^{3+} ion in Au nano-trench

We investigated the position, polarization dependent LDOS of an Au nano-trench through 3D-FDTD simulations. By taking the ratio of total emission power from the dipole source in an Au nano-trench to that in uniform SiO_2 media, we calculated the enhancement factor of total decay rate. We scanned the position of a dipole source to obtain the spatial profile of total decay rate enhancement as in Supplementary Figure 4a to c. The *z*-position of the dipole source is at the anti-nodal plane (z=0) and the interval length for the *x*- and *y*-scanning is set to 10 and 50 nm, respectively.

The total decay rate enhancement profile for the *x*-polarized dipole in Supplementary Figure 4a well reflects the spatial mode profile of $|E_x|^2$ in Supplementary Figure 4d. It is almost constant along the *x* direction and nearly cosinusoidal along the *y* direction. At the center (x=y=0), the maximum decay rate was determined as γ_{max}^{tot} =200γ_0^r, where γ_0^r is the intrinsic radiative decay rate of the dipole in uniform SiO_2 media. The arithmetic averaged decay rate (=$<\gamma^{tot}>$) was calculated to be 0.59γ_{max}^{tot}.

For the perpendicularly oriented dipoles in Supplementary Figure 4b and c, the arithmetic average decay rate is 0.036γ_{max}^{tot} and 0.16γ_{max}^{tot}, respectively and they barely contribute to the emission enhancement. Consideration of the light extraction efficiency and collection efficiency reveals that the contributions of the *y*- and *z*-oriented dipoles are only 0.0125 and 0.0128 of that of the *x*-polarized dipole. The experimental data in Figure S4e shows clear feature of the dominance of the *x*-polarization from F490 sample; it fits well to cosine squared function (red dashed-dot curve). The extinction ratio of the x-polarized intensity to that of the *y*-polarized one is measured to be 105. Therefore, we might omit the contributions of the *y*- and *z*-oriented dipoles when establishing multi-exponential decay model in supplementary information 5.

Supplementary Notes 4 | Multi-exponential decay model and initial slope

When many emitters are distributed in a spatially inhomogeneous LDOS, each emitter decays at a different rate depending on its position $\mathbf{r}_i$. If we assume that all the emitters are initially in the excited state at t=0, the probability $U_i(t,\mathbf{r}_i)$ of finding an emitter at $\mathbf{r}_i$ in the excited state at time t is expressed as $U_i(t,\mathbf{r}_i) = exp[-\gamma(\mathbf{r}_i)t]$, where $\gamma(\mathbf{r}_i)$ is position-dependent total decay rate of the emitter. Taking the collection geometry of the measurement system and light extraction efficiency of the cavity into account, the measured PL intensity $f(t)$ should be expressed as $\sum\eta(\mathbf{r}_i)\cdot\eta_{ext}(\mathbf{r}_i)\cdot\hbar\omega(-dU_i/dt)$, where $\eta(\mathbf{r}_i)$, $\eta_{ext}(\mathbf{r}_i)$, and $\hbar\omega(-dU_i(t,\mathbf{r}_i)/dt)$ are the collection efficiency, light extraction efficiency, and instantaneous power generated by an emitter at $\mathbf{r}_i$, respectively. The summation is over all the emitters and we consider the *x*-polarization only since the contributions of the *y*- and *z*-polarization are negligible as discussed in supplementary information 3.

If one see Figure 4a and Supplementary Figure 5, both of $\eta_{ext}(\mathbf{r}_i)$ and $\eta(\mathbf{r}_i)$ are nearly-independent of the emitter position. Under these conditions, the measured PL intensity can be expressed simply as $f(t)=\eta\cdot\eta_{ext}\cdot\hbar\omega\cdot\sum(-dU_i/dt)$. Then, the initial slope of the measured PL intensity at t=0, $[(df(t)/dt)/f(t)]_{t=0}$ becomes –$[\sum\gamma^2(\mathbf{r}_i)]/[\sum\gamma(\mathbf{r}_i)]$, which does not reflect the fastest decaying component only but also the other components in the ensemble of emitters.

Supplementary Notes 5 | Calculation of relative initial slope and relative radiant flux

In order to support the validity of experimentally determined relative initial slope and relative average radiant flux in Figure 3b and c, we calculated the corresponding quantities by 3D-FDTD method. In the experiments, what we measure is the total decay rate; $[\gamma^{tot}]_{exp} = F_P\gamma_0^r+\gamma_{d\text{-}d}^{loss}+\gamma_0^{nr}$, where F_P is Purcell factor by the cavity resonance and $\gamma_{d\text{-}d}^{loss}$ is the rate of Joule loss due to the dipole-dipole interactions between the emitter and metal [2, 3], respectively. Given the known values of γ_0^r and γ_0^{nr} from Supplementary Discussion 2 and the calculated mode profiles from Supplementary Discussion 3, we have calculated $(F_P\gamma_0^r+\gamma_{d\text{-}d}^{loss})$ for all positions, from which it is then straightforward to calculate the value of the initial slope, given by $[\sum\gamma^2(\mathbf{r}_i)]/[\sum\gamma(\mathbf{r}_i)]$. We find that the value of initial slope is related to the maximum Purcell factor, achieved at the center of the cavity (x=y=0), simply as $[\sum\gamma^2(\mathbf{r}_i)]/[\sum\gamma(\mathbf{r}_i)]=0.84\gamma_{max}^{tot}$. Thus, knowing the relative initial slope enables us to accurately derive the maximum Purcell factor achieved in the cavity. The resulting calculated values for cavities of different lengths are summarized in Fig. 3b.

Knowing the enhanced radiation rates, we can directly calculate the expected enhancement in the far field radiation flux, provided that the differences in the extraction and collection efficiencies are known. Therefore, we calculate the extraction efficiencies for each cavity using FDTD. The results are summarized in Supplementary Table 1.

Using the collection geometry to calculate the collection efficiencies, it is then straightforward to transform the enhancement of radiation rates to expected enhancement of far-field radiant flux. The results are summarized in Fig. 3c. We also find that the actual far field flux, which is an ensemble average of all Er^{3+} ions in the cavity, is simply related to the maximum enhanced radiant flux at the center of the cavity simply as $<\eta_{ext}\gamma^{tot}> = 0.58\eta_{ext}\gamma_{max}^{tot}$. Thus, an experimentally measured average radiant flux enhancement of 54 corresponds to 93-fold (54/0.58) enhancement of radiant flux at the mode maximum.

Supplementary Notes 6 | Pumping intensity for reaching saturation regime

For fair comparison of the radiant fluxes of spontaneous emission, all the samples should be pumped up to the saturation level such that all the Er^{3+} ions are excited and ready to decay through spontaneous emission. In our experiment, the InGaAs laser diode is able to supply the maximum pump intensity of 86 $mW\mu m^{-2}$, which is ~2,000 times of the saturation pump intensity of Er^{3+} ions [4].

In Supplementary Figure 6, the measured normalized radiant fluxes are shown as a function of pump intensity. In both cases, saturation characteristics are observed. At a normalized radiant flux of 0.5 (dotted line), the measured pump intensity of the F490 sample is ~15 times stronger than that of the control sample where Er-doped layer interfaces flat Au surface, which agrees with the prediction of three-level rate equations [5].

Supplementary Notes 7 | Near-to far-field transformation

The far-field profile contains the directional property of radiant flux. When it is integrated over the collection cone of given N. A. of the objective lens and normalized to that integrated over total solid angle, we obtain the collection efficiency. We employed the near-to far-field (NTFF) transformation method to obtain far-field information using the near-field profile calculated by 3D-FDTD simulations [6, 7].

We calculated the phasor of the electromagnetic fields over the surface of the enclosure (red dashed lines) which includes the dipole source and the Au nano-trench (Supplementary Figure 7a). We transform the near-field phasor to the corresponding far-field distributions in Supplementary Figure 7b. Here, we used a simple mapping defined as $(x, y) = (\theta\cos\varphi, \theta\sin\varphi)$ to project far-field profiles from spherical coordinate system to 2-D Cartesian coordinate system (Supplementary Figure 7c).

Supplementary Methods

| Fabrication of Au nano-trench structures

Au nano-trench structures were fabricated through a series of processes as depicted in Supplementary Figure 3. On a bare quartz wafer, a 200-nm-thick SiO_2 slab containing a 40-nm-thick Er-doped SiO_2 layer was deposited by multi-target, reactive ion-beam sputtering method. A chromium etching mask was formed by electron beam lithography and electron beam evaporation (e-beam evaporation), followed by a liftoff process. The SiO_2 slab was dry-etched by reactive ion beam etching (RIE), thus forming a thin and long rectangular mesa that is shown in Fig. 1a. Finally, the whole film was covered by an optically thick (1 μm) Au layer, thus inverting the original SiO_2 fin into a trench in the Au layer. Note that as the Au layer thickness is much larger than the original height of the fin, the Au layer effectively forms a continuous backlayer. The dimensions of the trench can be accurately controlled by the etching process, while the location of the Er-doped SiO_2 layer can be controlled by the deposition process.

Supplementary References

1. Snoeks, E., Lagendijk, A., and Polman, A. Measuring and Modifying the Spontaneous Emission Rate of Erbium near an Interface. *Phys. Rev. Lett.* **74**(13), 2459 (1995).

2. Chance, R. R., Miller, A. H., Prock, A., and Silbey, R. Fluorescence and energy transfer near interfaces: The complete and quantitative description of the Eu^{+3}/mirror systems. *J. Chem. Phys.* **63**(4), 1589 (1975).

3. Yu, N., Belyanin, A., Bao, J., and Capasso, F. Controlled modification of erbium lifetime by near-field coupling to metallic films. *New J. Phys.* **11**, 015003 (2009).

4. Navarro-Urrios. *et al.* Optically active Er^{3+} ions in SiO_2 codoped with Si nanoclusters. *J. Appl. Phys.* **106**, 093107 (2009).

5. Becker, P. C., Olsson, N. A., and Simpson, J. R. Erbium-Doped Fiber Amplifiers Fundamentals and Technology. *Academic Press, San Diego, CA* (1997).

6. Lee, E. K., Song, J. H., Jeong, K. Y., and Seo, M. K. Design of plasmonic nano-antenna for total internal reflection fluorescence microscopy. *Opt. Express* **21**(20), 23036 (2013).

7. Demarest, K., Huang, Z., and Plumb, R. An FDTD near-to far-zone transformation for scatters buried in stratified grounds. *IEEE Trans. Antenna Propagat.* **44**, 1150 (1996).
